# Photo–Thermally Sequentially Responsive Shape Memory Polymers Based on Side–Chain Azobenzene–Functionalized Epoxy

**DOI:** 10.3390/polym18151902

**Published:** 2026-08-03

**Authors:** Jinxiong Wen, Xianhao Mao, Shaojun Chen, Yuanyuan Li, Zhiwen Tu, Huilin Lai, Haitao Zhuo

**Affiliations:** 1 College of Materials Science and Engineering, Shenzhen University, Shenzhen 518053, China; wenle9451@163.com (J.W.); mxh18738569725@gmail.com (X.M.); 18944630307@163.com (Y.L.); 17875541747@163.com (Z.T.); 19914816496@163.com (H.L.); 2 College of Chemistry and Environment Engineering, Shenzhen University, Shenzhen 518053, China

**Keywords:** shape memory polymer, photo–thermally sequentially responsive, azobenzene

## Abstract

To address the critical limitations of traditional photothermal–responsive epoxy–based shape memory polymers (ESMPs), such as poor compatibility and cyclical instability, a series of side–chain azobenzene (Azo)–functionalized linear ESMPs (EPDm) were successfully synthesized via chemical bonding. Highly rigid 4–aminoazobenzene (Azodm) units were covalently integrated into the Bisphenol A–type epoxy backbone as functional photo–responsive side chains. Systematic characterizations confirmed that the glass transition temperature (T_g_) of the EPDm polymers could be precisely tuned from 41.53 °C to 53.26 °C by adjusting the Azodm content. Benefiting from the proposed homogeneous covalent architecture, the EPDm films exhibited superior photothermal sequential behavior and remarkable shape memory stability despite a slight reduction in tensile strength (≈10–12 MPa). Under 365 nm UV irradiation, the pre–stretched EPDm_10_ specimen achieved a rapid macroscopic bending angle of over 150° within 15 s, driven exclusively by the *trans*–to–*cis* molecular photoisomerization of the Azo side chains well below the matrix T_g_. Furthermore, standard shape memory cycles demonstrated excellent shape fixity (R_f_ > 98%) and recovery (R_r_ > 95%) ratios, alongside exceptional anti–fatigue performance with minimal strain variance (≈5%) over consecutive cycles. This work establishes a robust and high–efficiency molecular design strategy for photo–thermally sequentially responsive shape memory polymers, offering immense potential for smart actuators and flexible electronics.

## 1. Introduction

Shape memory polymers (SMPs) are smart macromolecular materials capable of undergoing reversible shape transitions under external stimuli, e.g., heat [1], light [2], electric fields [3], magnetic fields [4], or humidity [5]. Driven by their stimulus–responsiveness and 4D printing advancements, SMPs show immense potential in aerospace [6], biomedicine [7], and soft robotics [8]. Specifically, light–responsive SMPs enable the remote and localized programming of material geometry and mechanical behavior via optical stimuli [9,10,11,12], making them indispensable for advanced applications like soft robotics and intelligent actuation [13,14,15].

Despite the intrinsic merits of light–responsive SMPs, single–stimulus systems fundamentally suffer from restricted actuation output and poor cyclic endurance [16,17,18]. To address these impediments, multi–responsive shape memory polymer composites (SMPCs)—specifically photothermal–responsive systems—have been widely developed. Typically, conventional photothermal SMPs incorporate infrared (IR)–absorbing agents (e.g., polydopamine [19,20], graphene, or carbon nanotubes [21,22]) via physical blending [23,24]. These approaches still predominantly rely on doping with inorganic photothermal absorbers or organic dyes, which are highly prone to inducing macroscopic phase separation and filler agglomeration within the polymer matrix. This resulting interfacial incompatibility not only compromises the mechanical integrity of the composites but also exacerbates cyclic instability and diminishes actuation precision in complex operational environments [25]. Compounding these issues, organic dyes inherently suffer from poor stability [26].

In contrast to infrared photo–responsive systems relying on inorganic photothermal fillers, ultraviolet (UV) photo–responsive SMPs achieve shape alteration through photochemical reactions (e.g., photoisomerization) of photosensitive groups (e.g., azobenzene [27,28,29] or cinnamic acid [30,31]). By introducing this mechanism into thermally induced SMPs, step–wise deformations driven independently by light and heat can be achieved. This filler–free, purely organic design circumvents inherent inorganic filler issues such as aggregation, poor interfacial compatibility, and mechanical embrittlement, thereby preserving the material’s inherent flexibility and optical transparency [32,33]. Furthermore, this mechanism enables the orthogonalization of driving stimuli. Light drives deformation and fixation below the thermal transition threshold, while heat independently triggers shape recovery. Photo–thermally sequentially responsive shape memory behavior was used to describe this dual–stimulus system (UV photochemical deformation and subsequent thermal shape recovery) [27]. This effectively avoids accidental recovery caused by thermal diffusion in conventional infrared–responsive SMPs, ensuring the material remains thermodynamically frozen during photo–induced deformation to improve deformation precision and operational stability [34].

Azobenzene is one of the core components in such systems, undergoing reversible *trans*–to–*cis* photoisomerization under UV/visible light or thermal stimuli to govern macroscopic behaviors [35,36,37]. However, some traditional designs such as simple physical blending and side–chain modification frequently face issues like poor compatibility and cyclic instability. For instance, Shen et al. [38] fabricated a UV–thermal sequentially responsive SMP by physically blending azobenzene monomers and POSS into EVA. Although shape fixation was achieved, the physical blending nature inevitably creates weak interfacial zones, leaving the system susceptible to phase separation and stress concentration during long–term use. Similarly, Li et al. [39] grafted terminal azobenzene (TABA) onto a polyurethane network; while rapid actuation was achieved, the introduction of excessive TABA disrupted the inherent hydrogen bonding of the system and induced a chain lubrication effect, causing the shape recovery ratio to drop below 70%. Furthermore, while Mao et al. [27] advanced processability via 4D printing of azobenzene pre–polymers, balancing processability with the structural integrity of the polymer network remains a major challenge.

Unlike these strategies, this study introduces a highly stable, covalently integrated, purely organic architecture based on shape memory epoxy resins (EPD series). Specifically, side–chain functionalization of the EPD matrix was achieved by directly covalently bonding *p*–aminoazobenzene as a reactive monomer onto the polymer backbone. This strategy effectively eliminates interfacial mismatches and plasticization effects, ensuring robust cyclic stability and precise step–wise actuation of the material. We systematically investigated the regulatory role of this covalently bonded azobenzene architecture on the photo–thermally sequential responsiveness and shape memory properties of the epoxy shape memory polymers (ESMPs). Furthermore, their molecular structures, along with thermal, mechanical, and photo–responsive properties, were characterized (via FT–IR, ^1^H–NMR, GPC, DSC, TGA, and DMA). This work aims to provide a solid theoretical foundation for the development of high–efficiency, intelligent shape memory materials.

## 2. Synthesis and Characterization

### 2.1. Raw Materials

Bisphenol A–type epoxy resin (E–51, Industrial grade) and diethylene glycol amine (DGA, 98%) were purchased from Shanghai Macklin Biochemical Co., Ltd., Shanghai, China. Polypropylene glycol diglycidyl ether (PPGDE, Mn~500), Azobenzene (Azo, 98%), and *p*–aminoazobenzene (Azodm, 98%) were purchased from Shanghai Aladdin Biochemical Technology Co., Ltd., Shanghai, China.

### 2.2. Preparation of EPD Series

**Figure 1:**
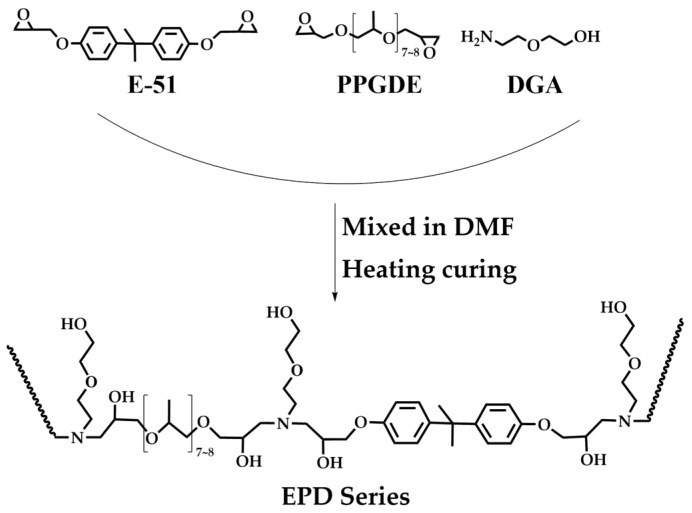
EPD synthesis route diagram.

E–51, PPGDE, and DGA were weighed into a 50 mL round–bottom flask according to the stoichiometric ratios specified in Table 1. The synthetic route is illustrated in Figure 1. A magnetic stirring bar was placed inside the flask to blend the mixture thoroughly, followed by ultrasonic degassing. This “stirring–degassing” cycle was repeated three times. Subsequently, the mixture was cast into a polypropylene plastic box and soft–baked in a constant–temperature blast drying oven at 60 °C for 2 h to evaporate most of the solvent, followed by a high–temperature drying step at 100 °C for another 2 h to ensure complete solidification. The resulting epoxy resin films were transparent, homogeneous, and bubble–free. These matrix materials were designated as the EPDx series, where x represents the molar percentage of E–51 within the system.

### 2.3. Preparation of EPDm Series

**Figure 2:**
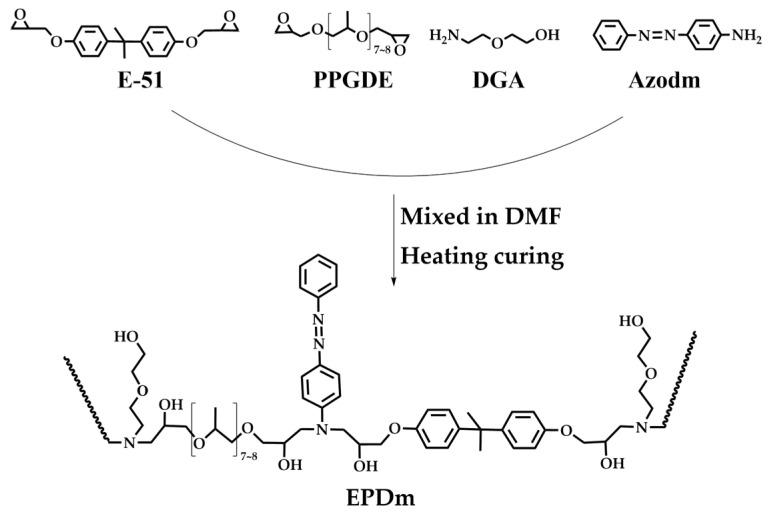
EPDm synthesis route diagram.

The EPD_94_ was utilized as the ESMP matrix to synthesize the azobenzene–functionalized side–chain polymers (EPDm). The amino groups of Azodm can react with epoxy groups. Therefore, an increase in the dosage of Azodm necessitates a corresponding reduction in the amount of DGA. Otherwise, a stoichiometric excess of amino groups would lead to crosslinking, resulting in the formation of insoluble matter. The synthetic route is illustrated in Figure 2. Initially, E–51, PPGDE, DGA, and Azodm were weighed into a flask according to the proportions specified in Table 2, followed by the addition of 50 mL of DMF as a solvent for dilution. A magnetic stirring bar was placed inside, and the flask was immersed in an oil bath at 60 °C with continuous stirring for 2 h to allow a thorough reaction. Subsequently, the temperature of the oil bath was elevated to 100 °C to continue the reaction for another 2 h. The resulting mixture was cast into a polypropylene plastic box and subjected to vacuum drying in a vacuum oven at 60 °C for 2 h to remove the DMF solvent. The obtained SMPC films were transparent, homogeneous, and bubble–free, which were designated as the EPDm_x_ series composites, where x represents the molar percentage of Azodm within the system.

### 2.4. Characterization

The chemical bondings in the materials were studied by Fourier transform infrared spectrometry (FT–IR, Nicolet 760, Madison, WI, USA) in attenuated total reflection (ATR) mode under a spectral resolution of 4 cm^−1^ with 10 scans for each measurement.

The crystal structures were identified by X–ray diffraction (XRD, Miniflex600, Rigaku, Tokyo, Japan) at a scan rate of 10°/min by scanning 0.5 mm thick splines or powder.

Further identification of bonding was carried out by nuclear magnetic resonance (NMR, Avance III 500 MHz, Bruker, Karlsruhe, Germany) spectrometry operating at a proton frequency of 500 MHz using samples dissolved in deuterated dimethyl sulfoxide (DMSO–d_6_) with tetramethylsilane (TMS) as an internal standard under spectral resolution of 0.1 Hz with 32 scans accumulated for each measurement.

The thermal properties were assessed by differential scanning calorimetry (DSC–25, TA Instruments, New Castle, DE, USA) under nitrogen purging, spanning temperatures from −40 °C to 200 °C, and a heating rate of 10 °C/min.

The sample mass loss rate as a function of temperature was determined by thermogravimetric analysis (TGA, TGA55, TA Instruments, New Castle, DE, USA) using samples (10–20 mg) dried at 80 °C for 1 h and tested in a 40 mL/min nitrogen atmosphere at a heating rate of 10 °C/min from room temperature to 600 °C.

The storage modulus and tan δ as a function of temperature were determined by dynamic mechanical analysis (DMA, Q800, TA Instruments, New Castle, DE, USA) using a multi–frequency strain mode at 10 Hz. The samples were initially stabilized at −50 °C for 5 min and then tested at a heating rate of 3.0 °C/min up to 150 °C for real–time data recording.

The shape memory performance was evaluated using the same DMA (Q800, TA Instruments, New Castle, DE, USA) in a controlled force mode with a preload of 0.001 N and a force track of 125%. The test cycle involved heating the sample to 60 °C at 10 °C/min (held for 5 min), loading to 0.3 N at 0.1 N/min (held for 5 min), cooling to −20 °C at 10 °C/min for shape fixing (held for 5 min), unloading to 0 N at 0.1 N/min (held for 5 min), and finally reheating to 60 °C at 10 °C/min for shape recovery.

Tensile tests of each material were performed on a universal testing machine (CMT 4204, Sans, Shenzhen, China) to determine the tensile strength and elongation at break with a 20 KN sensor according to GB/T 1040.1-2018 [40]. The splines were then stretched at different elongations (50%, 100%, 150%, 200%, and 250%) at room temperature of 25 °C, with a stretching speed of 20 cm/s.

The molecular weights and distributions were characterized by LC98IIRI gel chromatography (Beijing Temperature Analysis Instrument Technology Development Co., Ltd., Beijing, China) using DMF as the eluent.

The UV–Vis absorption properties were studied by UV–vis spectrometry (Lambda 950, PerkinElmer, Waltham, MA, USA) using samples dissolved in DMF to prepare a 0.005 mol/L solution, scanned at 1 nm/s from 300 to 600 nm after different durations of UV irradiation.

Photoresponsive bending (θ_1_) was evaluated using a UV source (MUA–165, λ = 365 nm) inside a dark, dust–free enclosure. Stretched rectangular specimens (≈30 × 5 × 1 mm^3^) were mounted horizontally, 5 cm below the light source center. Bending angles were recorded every 5 s over a 30 s exposure, with results averaged across three replicates. To compensate for fixed–illumination limits, a step–wise strategy was deployed: the UV source was positioned at θ = 90° for θ_1_ < 90° and manually translated toward 180° once θ_1_ ≥ 90° to sustain exposure until termination. The testing procedure is illustrated in Figure S4.

## 3. Results and Discussion

### 3.1. Structure Analysis

**Figure 3:**
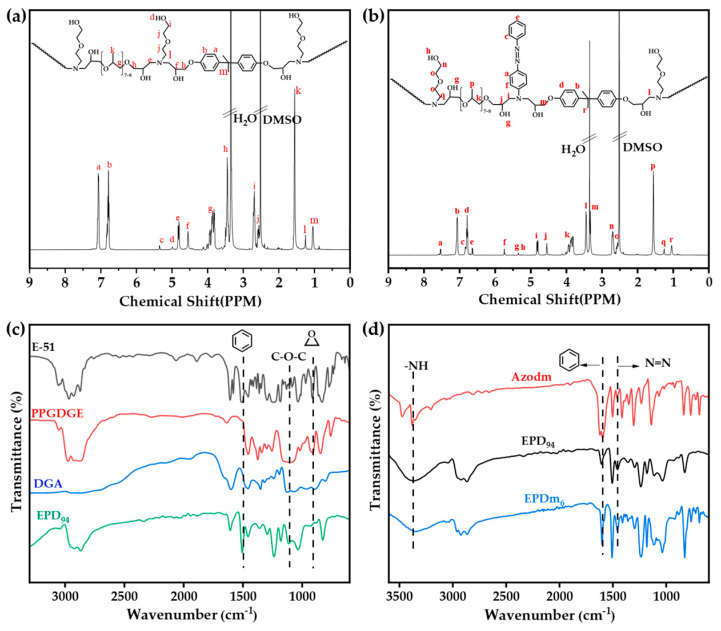
^1^H–NMR spectrum of (**a**) EPD_94_ and (**b**) EPDm_6_; FT–IR spectra (**c**,**d**) of raw materials, EPD_94_ and EPDm_6_.

To confirm the successful covalent incorporation of the photo–responsive monomer Azodm into the polymer backbone, ^1^H–NMR spectroscopy was performed (Figure 3a,b). Compared to the pristine EPD_94_ matrix, the primary distinction of EPDm_6_ lies in the partial replacement of DGA with Azodm, which serves as a reactive chain–extending monomer. In the ^1^H–NMR spectra of EPDm_6_, several additional proton signals originating from the Azodm moiety are distinctly observable, specifically at positions a, c, e, f, and g, corresponding to the various proton environments on the aromatic rings. Furthermore, the chemical shifts in the main matrix proton signals remain highly consistent between EPD_94_ and EPDm_6_. Rather than indicating a similarity in macroscopic properties, this spectral consistency confirms that the introduction of Azodm proceeds via the intended specific reaction pathways and does not disrupt the fundamental epoxy–amine linkage mechanism of the backbone.

FT–IR spectroscopy was employed to monitor the ring–opening reaction and preliminarily verify the chemical structure of the resulting polymers. As shown in Figure 3c,d, the characteristic infrared absorption peaks of the EPD matrix and the Azodm monomer are primarily located at 2830, 1500, 1100, and 920 cm^−1^. Specifically, the absorption peaks at 1500 and 1100 cm^−1^ are assigned to the C=C vibration of the benzene ring in E–51 and the C–O–C stretching vibration in PPGDE, respectively. The peak at 920 cm^−1^ corresponds to the characteristic absorption of the epoxy groups present in both E–51 and PPGDE. Notably, this epoxy absorption peak almost completely disappears in EPD_94_. Concurrently, the disappearance of the characteristic –NH peak from Azodm is also observed. The simultaneous consumption of both epoxy and amino groups provides direct evidence for the proposed epoxy–amine ring–opening mechanism. Combined with the appearance of the N=N stretching vibration peak (1450 cm^−1^) and the more pronounced benzene ring peaks in EPDm_6_, these results verify that Azodm successfully acts as a reactive monomer to form covalent bonds within EPDm series. Additionally, the well–retained C–O–C and aromatic vibration features demonstrate that the intrinsic epoxy backbone remains structurally intact after grafting. Overall, the FT–IR results verify the successful covalent embedding of photo–responsive azobenzene units into the polymer matrix, providing a structural foundation for subsequent UV–triggered trans–cis photoisomerization and macroscopic bending.

GPC was performed using DMF as both the solvent and the eluent. The molecular weights and corresponding distributions for the EPD_94_ and EPDm_6_ samples are summarized in Table S1. Specifically, EPD_94_ exhibits a dispersity of 1.096, while EPDm_6_ maintains a comparably low value of 1.108. This narrow molecular weight distribution of EPD_94_ and EPDm_6_ originates from the well–controlled stoichiometry and homogeneous solution polymerization adopted in this work, which differs from conventional bulk step–growth epoxy curing. No significant difference is observed in the molecular weight distributions between the two polymers. This suggests that the incorporation of the Azodm monomer does not appear to induce evident branching, cross–linking, or uncontrolled polymerization, thus preserving the linear architecture of the epoxy backbone. As a result, the linear backbone of the pristine EPD matrix remains well preserved after Azodm functionalization, providing an essential basis for the high cyclic stability and mechanical homogeneity of these photo–thermally sequentially responsive shape memory polymers. Compared with EPD_94_, EPDm_6_ shows a slight decrease in Mn due to the partial replacement of long–chain DGA with small rigid Azodm molecules, which foreshadows the slight decline in tensile strength discussed in the subsequent mechanical performance section.

**Figure 4:**
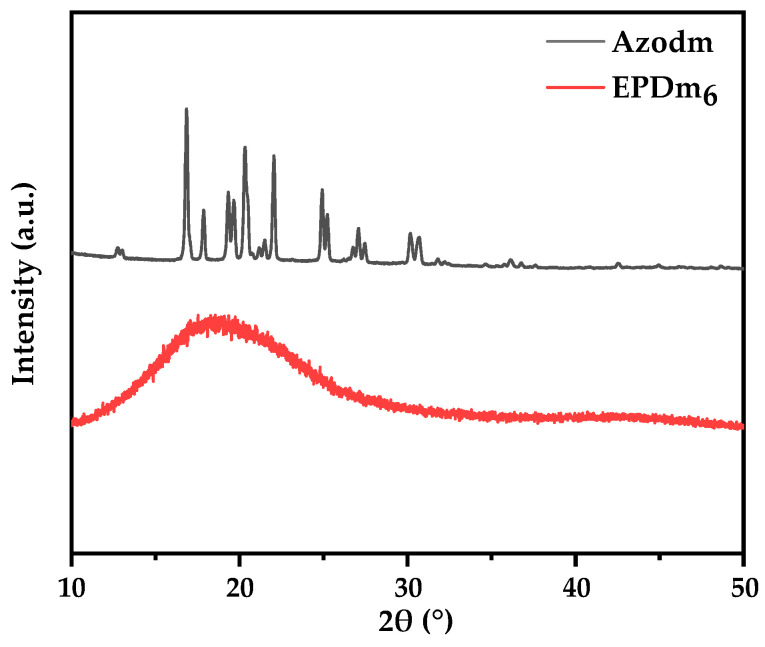
XRD patterns of Azodm monomer and EPDm_6_.

Figure 4 presents the crystal structure information of the pristine Azodm and EPDm_6_. As clearly observed from the spectra, the pristine Azodm exhibits typical crystalline diffraction characteristics, evidenced by a series of sharp and high–intensity diffraction peaks, which indicate a highly ordered crystalline structure. In contrast, the XRD pattern of EPDm_6_ displays only a single broad amorphous halo, indicating a disordered atomic arrangement within the material. This demonstrates that Azodm loses its liquid crystalline characteristics upon reacting with the EPD matrix. Specifically, when Azodm is incorporated into the epoxy main chain as side chains via covalent reactions, the ordered arrangement of the liquid crystalline molecules is disrupted by the random segments of the epoxy resin. It is proposed that the steric hindrance imposed by the epoxy segments restricts the rotational and translational freedom of the Azodm molecules, which may contribute to destroying the ordered environment essential for the crystallization of liquid crystalline molecules.

In addition, EPD and EPDm samples were cryogenically fractured in liquid nitrogen, and their cross–sectional morphologies were further characterized via SEM. Figure S2 shows a smooth, pore–free brittle fracture surface for EPD_99_. As PPGDE loading rises, dense fine wrinkles and fibrillar features emerge, increasing fracture surface roughness. This morphology shift reveals higher energy absorption during failure, corresponding to a transition from brittle to ductile fracture. For EPDm specimens, SEM of cryo–fractured cross–sections (Figure S3) confirms no phase separation. As Azodm content increases from EPDm_2_ to EPDm_10_, fracture surfaces develop more prominent layered textures and abundant fibrous structures. These results further confirmed that Azodm monomers react with epoxy groups via epoxy–amine ring–opening and are covalently tethered onto the epoxy main chain as molecular side groups, rather than physically blended as a discrete secondary filler phase. They also matched the featureless amorphous broad halo in XRD.

### 3.2. Thermal Performance Analysis

**Figure 5:**
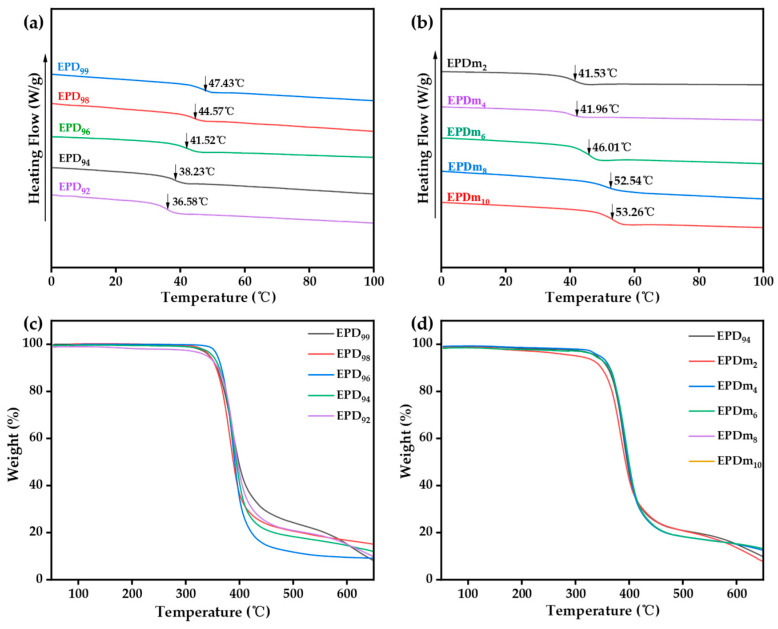
DSC secondary warming curves of (**a**) EPD series and (**b**) EPDm series. TGA curves of (**c**) EPD series and (**d**) EPDm series.

DSC was performed on the EPD and EPDm series samples to investigate the effects of increasing PPGDE content and incorporating Azodm monomers on the phase transition behavior of the ESMP composites. In particular, the glass transition temperature (T_g_) was determined from the second heating curves. As illustrated in Figure 5a, with the gradual increase in PPGDE content in the EPD system, the T_g_ decreases progressively from 47.43 °C (EPD_99_) to 36.58 °C (EPD_92_). This indicates that PPGDE acts as a flexible segment for tuning the T_g_ within this system. In addition, as shown in Figure 5b, the T_g_ of EPDm series rises linearly from 41.53 °C (EPDm_2_) to 53.26 °C (EPDm_10_) as Azodm loading increases. A plausible molecular interpretation for this upward T_g_ trend is that rigid azobenzene side chains introduce steric hindrance that limits the segmental mobility of epoxy backbones. Consequently, Azodm serves a dual purpose in this system: acting as a side–chain functionalizing amine to partially substitute DGA, while simultaneously imparting photo–responsive properties.

TGA was conducted on the EPD and EPDm series samples to investigate the respective impacts of increasing PPGDE and Azodm content on the thermal stability of the ESMP composites. As illustrated in Figure 5c,d, both the EPD system and the EPDm composite system exhibit excellent thermal stability. The EPD system features an onset decomposition temperature around 350 °C and completes its degradation process near 600 °C. Compared to the EPD_94_ matrix, the incorporation of Azodm induces no significant alterations in the thermal stability of the SMPCs. Notably, only the EPDm_2_ sample, which contains the lowest Azodm content, exhibits slight premature thermal decomposition. It is hypothesized that this behavior is ascribed to the structural similarity between Azodm and DGA; however, owing to the lower reactivity of Azodm, we suggest that shorter polymer chains might form initially without a homogeneous reaction. With the incremental increase in Azodm content, the chain segment distribution within the EPDm series polymers is presumed to become more uniform, thereby leading to a consistent weight loss behavior.

### 3.3. Mechanical Properties

**Figure 6:**
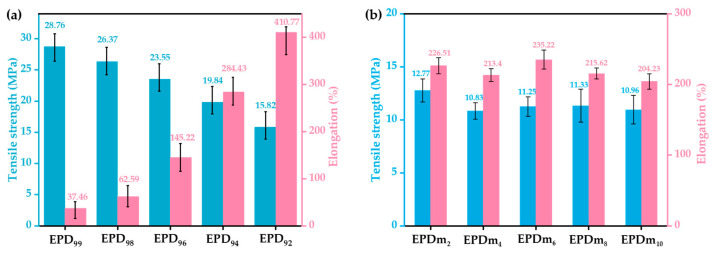
Tensile strength and elongation at break: (**a**) EPD series and (**b**) EPDm series.

Tensile tests were conducted on the EPD and EPDm system specimens at a room temperature of 25 °C using a universal testing machine with a crosshead speed of 10 mm/min to investigate the effects of PPGDE and Azodm content on the mechanical properties of the composites. The tensile results are presented in Figure 6a,b. Since PPGDE serves as the soft segment in the SMPCs, the toughness of the specimens improves with increasing PPGDE content, as evidenced by the significant increase in elongation at break from 37.46% to 410.77%. Conversely, the substantial amount of benzene rings in the rigid E–51 framework provides strong strain resistance, thereby imparting high tensile strength; specifically, EPD_99_ reaches a tensile strength of 28.76 MPa. As the soft–segment content increases in EPD_94_, the tensile strength decreases to 19.84 MPa. Regarding the EPDm system, with the incremental increase in Azodm content, the maximum elongation at break of the EPDm series specimens decreases gradually from 226.51% to 204.23%, while the tensile strength concomitantly declines from 12.77 MPa to 10.96 MPa. The overall mechanical properties exhibit minimal fluctuations, which is likely attributed to the homogeneous distribution of Azodm within the epoxy backbones enabled by chemical bonding. Compared to the EPD_94_ matrix, the films fabricated from the EPDm series materials show a slight reduction in tensile strength. This indicates that when Azodm is introduced as a functional chain–extending monomer to partially replace DGA, its reinforcing efficiency is inferior to that of the homogeneous linear epoxy chains formed by DGA and E–51. Although Azodm possesses multiple benzene rings, it is incorporated as side chains onto the epoxy main chain, thus exerting a limited impact on the mechanical properties. This outcome corroborates the previously discussed GPC results; the slightly broader molecular weight distribution and the reduced Mn of the EPDm series suggest the presence of shorter chain segments within the system, ultimately leading to the decline in the tensile strength of the specimens.

### 3.4. Photo–Responsive Shape Memory Behaviors

**Figure 7:**
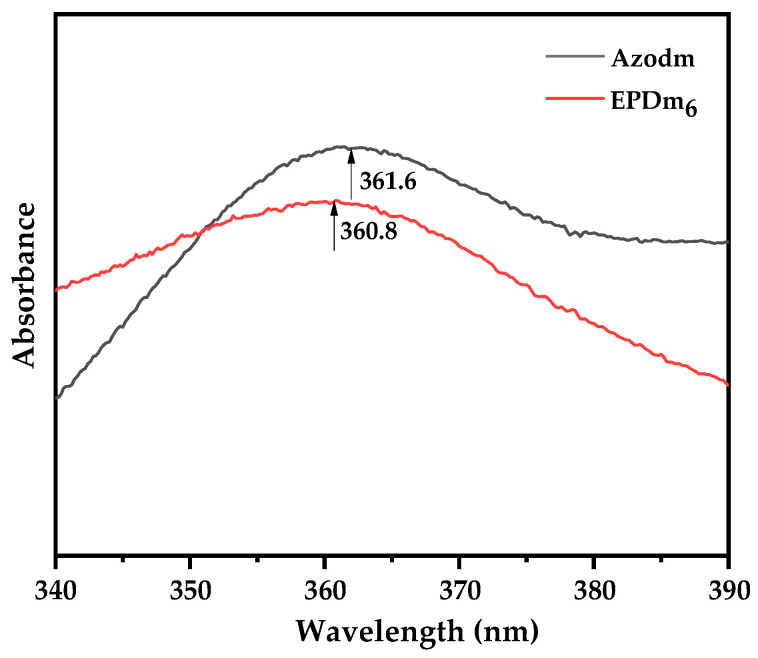
UV–Vis spectra of Azodm and EPDm_6_ in DMF solution.

To characterize the ultraviolet (UV) absorption, pristine Azodm and EPDm_6_ were separately dissolved in DMF to prepare solutions with a concentration of approximately 5 mg/mL, followed by UV–Vis spectrophotometry characterization. As illustrated in Figure 7, both samples exhibit a distinct absorption peak at 361 nm, demonstrating that the EPDm series can undergo a *trans*–to–*cis* isomerization under UV flashlight irradiation. The covalent bonding between Azodm and the epoxy backbone induces a minor blue shift in the photo–responsive wavelength of the Azo moieties. Nevertheless, the peak position aligns well with the nominal wavelength of 365 nm for the UV light source.

**Figure 8:**
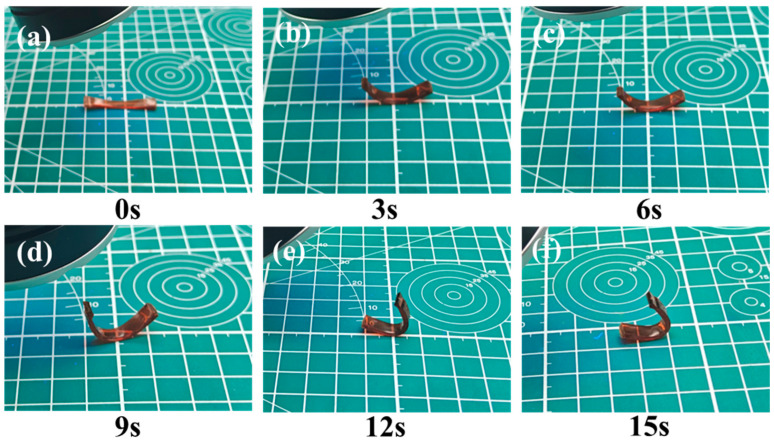
The bending process of the spline under UV irradiation from 0 to 15 s: (**a**) 0 s; (**b**) 3 s; (**c**) 6 s; (**d**) 9 s; (**e**) 12 s; (**f**) 15 s.

Notably, realizing such azobenzene–driven photo–thermally sequential shape memory properties require pre–stretching the material for orientation [27]. During this process, the polymer backbones and the covalently linked azobenzene side groups are compelled to align along the drawing direction, forming an oriented molecular chain structure [41,42]. This molecular orientation enhances the structural order of the photosensitive domains, enabling a cooperative photoisomerization of the azobenzene groups upon light irradiation and consequently altering the macroscopic shape of the material [43]. Therefore, specimens from the EPDm system with a thickness of 1 mm were employed to investigate their UV–responsive behaviors. Since most of the EPDm backbones can only sustain a maximum elongation of 200%, the EPDm_10_ specimen pre–stretched to a draw ratio of 200% was selected for the photo–responsive bending experiments. Mirroring the behavior of other EPDm backbones, the light–responsive Azodm moieties within EPDm also exhibit a photothermal conversion effect. The initial surface temperature of the specimen was recorded as 26.0 °C. Upon UV irradiation for 15 s, the photoinduced bending angle reached 153°, while the hotspot temperature rose to 35.1 °C. Considering that the T_g_ of EPDm_10_ is 53.6 °C, the peak temperature at the hotspot did not reach the shape memory effect (SME) switching temperature of the polymer matrix itself. Consequently, it can be inferred that the driving force behind the bending deformation of the EPDm_10_ specimen originates from the photoisomerization of the Azodm units attached as side chains to the molecular structure. Under UV illumination, the azobenzene molecules undergo *trans*–to–*cis* isomerization and molecular twisting, which subsequently drives the movement of the epoxy backbone, thereby guiding the pre–oriented specimen to bend toward the UV light source. The detailed bending response process and the corresponding temperature evolution at the hotspot are illustrated in Figure 8 and Figure S5, respectively.

**Figure 9:**
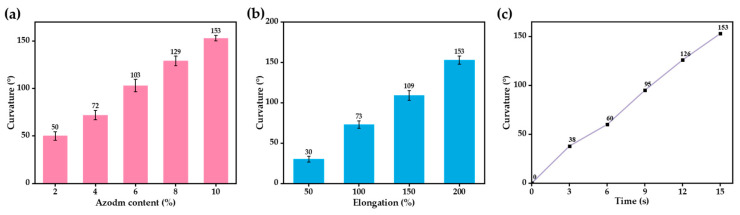
(**a**) Histogram of EPDm series spline under different Azodm content; (**b**) Histogram of EPDm_10_ spline under different tensile degree; (**c**) Curvature curve of EPDm_10_ spline under different UV–light times.

To quantitatively evaluate the factors influencing the UV photo–responsive behavior of the EPDm system, a systematic parametric analysis was conducted by varying one parameter at a time, specifically focusing on the Azodm content, pre–stretching draw ratio, and irradiation duration. The analytical results are presented in Figure 9a–c.

To investigate the effect of Azodm content on the UV–responsive bending behavior, EPDm series specimens with a thickness of 1 mm were pre–stretched to a draw ratio of 200%, and the variation in their bending angles was recorded under UV irradiation for 15 s. As illustrated in Figure 9a, with the increasing Azodm content, the bending angle of the EPDm specimens increases progressively, reaching a maximum of 153°. This trend can be attributed to the homogeneous covalent bonding between Azodm and the EPD matrix. Concurrently, as the content of the photo–responsive Azodm moieties increases, a greater number of Azodm units undergo *trans*–to–*cis* photoisomerization within the same temporal window. Consequently, this generates a stronger localized stress differential, leading to an enhanced actuation capability to induce macro–deformation and bend the specimens.

To investigate the effect of orientation degree, EPDm_10_ specimens with a thickness of 1 mm were subjected to tensile orientation at various draw ratios, and their UV–responsive bending angles were recorded, as depicted in Figure 9b. Under UV irradiation for 15 s, the bending angle of the EPDm_10_ specimens increases progressively with the increasing stretch ratio, reaching a maximum value of 153°. This behavior is attributed to the enhanced alignment of the liquid crystalline moieties at higher draw ratios, which yields a more ordered spatial arrangement of the Azodm units. Consequently, a larger fraction of liquid crystalline domains satisfy the Bragg diffraction conditions, which is proposed to lead to leading to highly efficient, cooperative photoisomerization within the 15 s temporal window. This synchronized molecular–level twisting successfully translates into the observed macroscopic bending deformation of the polymer specimens.

EPDm_10_ specimens with a thickness of 1 mm were pre–stretched to a draw ratio of 200% and subjected to UV irradiation for 15 s. The time–dependent evolution of their bending angles is depicted in Figure 9c. With the increasing irradiation duration, the Azodm moieties within the EPDm_10_ polymer backbones transition from the trans to the cis conformation, thereby driving the macroscopic bending deformation of the specimens. Mechanistically, the liquid crystalline Azodm units are covalently incorporated as side chains onto the rigid epoxy backbone. Upon UV light–induced photoisomerization, the *trans*–to–*cis* transition triggers a localized molecular volume expansion, which subsequently impels the bending of the matrix. Under UV illumination, the twisting of these side–chain Azodm molecules is proposed to effectively drive the cooperative movement of the epoxy main chain, thereby imparting a rapid photo–flexing response to the EPDm system.

Notably, the *trans*–to–*cis* isomerization of azobenzene introduces molecular free volume and disrupts local packing, a phenomenon known to cause a drastic decrease in local T_g_ in main–chain semicrystalline poly(azobenzene)s [44]. However, such massive T_g_ variations induced by conformational changes typically require specifically designed main–chain azopolymer architectures [45]. The active groups in our system are *p*–aminoazobenzene small molecules covalently integrated as side chains. While photoisomerization may cause a certain degree of local T_g_ depression, this local plasticization likely plays only an auxiliary role by lowering the energy barrier for sub–T_g_ deformation. As established by classical studies on glassy azobenzene networks [46], if the actuation were merely a photoplastic phenomenon dominated by T_g_ reduction, the material would macroscopically exhibit softening or stress relaxation rather than active movement. Therefore, the rapid, directional macroscopic bending observed in the EPDm system is primarily generated by steric hindrance and geometric shape changes.

### 3.5. Thermal Responsive Shape Memory Behaviors

**Figure 10:**
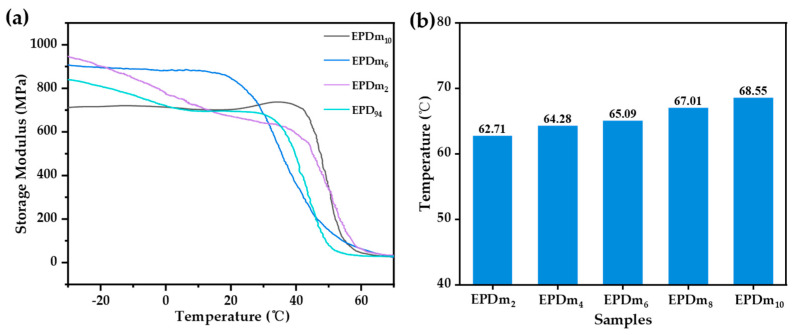
(**a**) Storage modulus and (**b**) temperature at peak Tan δ of EPDm series.

According to the experimental results presented in Figure 10, the EPDm system is in a glassy state within the low–temperature region below its T_g_, exhibiting a relatively high storage modulus (E′). As the temperature rises, the mobility of the polymer chains is enhanced, leading to a gradual decline in the storage modulus. When the temperature approaches the T_g_, a precipitous drop in the storage modulus is observed, signifying that the material undergoes a glass transition and enters the rubbery state, where the sharply increased polymer chain mobility results in a substantial reduction in the storage modulus. Concurrently, the loss factor (tan δ) reaches its maximum value at this temperature. The deviation between this peak temperature and the T_g_ determined via DSC characterization is within 15 °C, which lies well within a reasonable and acceptable range.

**Figure 11:**
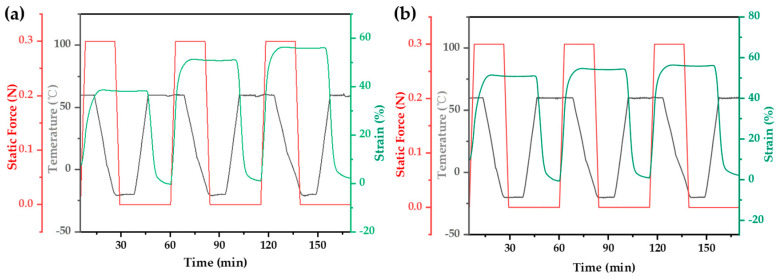
Shape memory cycling properties of (**a**) EPD_94_ and (**b**) EPDm_6_.

Figure 11a,b display the typical shape memory cyclic curves of EPD_94_ and EPDm_6_, respectively. Initially, the EPD_94_ specimen was subjected to three consecutive programming–recovery cycles. For a single–step SME cycle, the programming started with a heating stage, where the specimen was heated to 60 °C. Under an isothermal condition, a tensile force was applied and incrementally increased to 0.3 N until the deformation ceased, yielding a maximum strain of ε_0_ = 38.65% along the stretching direction. Subsequently, the specimen was cooled progressively to −20 °C while maintaining the static force at 0.3 N. During this cooling phase, slight thermal contraction occurred along the stretching direction; however, because the polymer chain mobility was suppressed at low temperatures, the specimen length became locked after the contraction. Once the specimen state stabilized, the post–contraction strain was recorded as ε_f_ = 38.12%. At this juncture, the tensile force was unloaded, resulting in no observable change in length due to the vitrified state of the specimen. Reheating the specimen to 60 shape recovery releases the internal entropic stress. Upon completion of the recovery process, the residual strain was recorded as ε_r_ = 0.22%. According to Equation (S6i,ii), the shape fixity ratio (R_f_) and shape recovery ratio (R_r_) of the EPD_94_ specimen for the first cycle were calculated to be 98.33% and 99.42%, respectively. During the second and third cycles, the ε_r_ from the preceding cycle represented the baseline value after complete recovery. Therefore, the calculation of the maximum strain for the subsequent cycle (ε_01_) necessitated subtracting the prior ε_r_; similarly, the corresponding fixed strain also required this correction. Consequently, the data were determined as ε_01_ = 51.08%, ε_f1_ = 50.57%, ε_r1_ = 0.85%, ε_02_ = 55.30%, ε_f2_ = 54.75%, and ε_r2_ = 1.14%. Based on Equation (S6iii,iv), the shape memory parameters were calculated as R_f2_ = 99.00%, R_r2_ = 98.32%, R_f3_ = 99.01%, and R_r3_ = 97.92%. Notably, a significant discrepancy in the maximum strain is observed among the three shape memory cycles, with ratios of (ε_r_ + ε_01_)/ε_0_ = 132.73% and (ε_r_ + ε_r1_ + ε_01_)/ε_0_ = 110.36%. Collectively, these ratios, together with R_f_ and R_r_, evaluate the cyclical stability of the shape memory performance. Although EPD_94_ exhibits excellent shape fixity and recovery ratios, the substantial deviation in ε_0_ indicates that during the initial stretching, internal spatial deformation and segment movement disrupt and redistribute the hydrogen bonding interactions. This structural reorganization gradually stabilizes after undergoing the cooling–heating cycles, as evidenced by the attenuated variation in the deformation peaks during the third cycle. Nevertheless, the cyclical stability of EPD_94_ still requires further optimization.

The EPDm6 specimens were tested using the same method, yielding ε_0_ = 51.32%, ε_f_ = 50.78%, ε_r_ = 0.30%, ε_01_ = 53.87%, ε_f1_ = 53.39%, ε_r1_ = 0.94%, ε_02_ = 56.57%, ε_f2_ = 55.75%, and ε_r2_ = 0.72%. Calculated using the same formulas, the results are R_f_ = 98.95%, R_r_ = 99.41%, R_f2_ = 99.11%, R_r2_ = 98.24%, R_f3_ = 98.55%, and R_r3_ = 98.71%. The corresponding strain ratios were determined to be (ε_r_ + ε_01_)/ε_0_ = 105.56% and (ε_r_ + ε_r1_ + ε_01_)/ε_0_ = 106.72%. Compared to the EPD matrix, EPDm_6_ maintains comparably good R_f_ and R_r_, while its shape memory cyclic stability is noticeably improved. Notably, the deviation in the maximum strain during consecutive cycles is significantly narrowed. This optimization is primarily attributed to the reduced dosage of DGA during the preparation of the EPDm series. Furthermore, the free Azodm within the system preferentially reacts with the epoxy groups during the heating stage, which is hypothesized to help suppress potential side reactions between the epoxy groups and hydroxyl groups. The small amount of remaining unreacted Azodm, acting as small molecules, might function as a molecular lubricant, thus exerting a negligible adverse impact on the overall shape memory performance.

**Figure 12:**
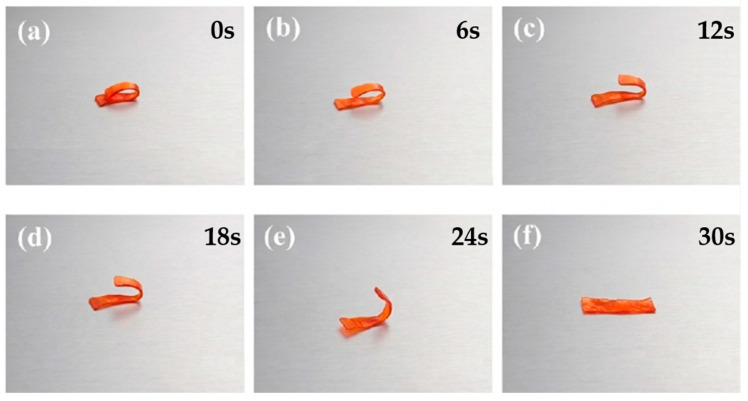
Thermal shape recovery process of the EPDm_10_ at 80 °C within 30 s: (**a**) 0 s; (**b**) 6 s; (**c**) 12 s; (**d**) 18 s; (**e**) 24 s; (**f**) 30 s.

The macroscopic shape recovery process of the specimens is presented in Figure 12. Upon heating for 30 s, the bending angle of the EPDm_10_ specimen recovers almost completely from the photoinduced deformation. This thermal treatment serves a dual purpose: it not only drives the material to trigger its intrinsic shape memory recovery process but also provides sufficient thermal energy to promote the back–isomerization of the Azodm units into the thermodynamically more stable trans conformation. Nevertheless, during this thermal recovery stage, the EPDm specimen also undergoes distinct thermal contraction along the original stretching orientation direction.

**Figure 13:**
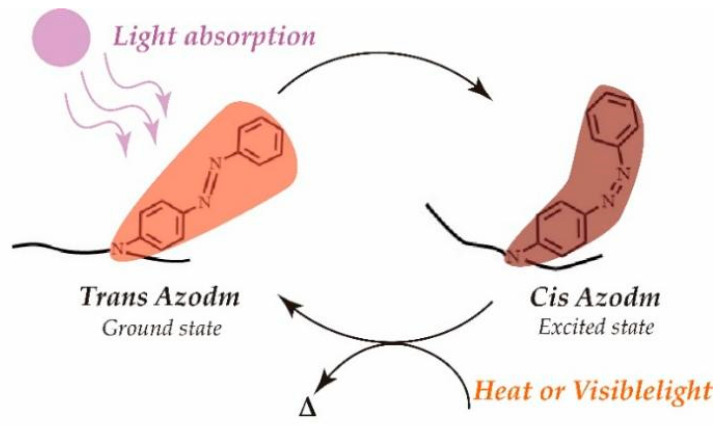
*Cis–trans* isomeric transformation mechanism of Azodm.

Therefore, this azobenzene–grafted epoxy polymer delivers orthogonal two–stage sequential actuation triggered separately by UV light and heat, termed photo–thermally sequentially responsive behavior. Light and heat act via independent molecular pathways, and the full cycle includes UV photochemical programming and thermal shape recovery. For full photo–thermally sequentially responsive tests, EPDm films are pre–stretched to 200% at room temperature to align epoxy backbones and covalently bound trans–azobenzene side chains along the tensile direction. 365 nm UV light converts ordered rod–like trans–Azodm into bent, bulky cis–Azodm. This structural change generates anisotropic stress at grafting sites. Collective twisting of aligned azobenzene units pulls epoxy chains, bending the film toward the UV source (EPDm_10_ bends over 150° in 15 s). At temperatures below Tg, the rigid glassy matrix traps metastable cis–Azodm, fixing the bent shape with R_f_ > 98%. Heating the deformed sample above bulk T_g_ triggers two simultaneous recovery processes. Epoxy chains regain mobility to release stored entropic strain and revert to their original conformation. Meanwhile, cis–Azodm thermally back–isomerizes to stable trans–Azodm as shown in Figure 13, removing UV–induced directional stress and fully restoring the initial shape.

## 4. Conclusions

In summary, we developed photo–thermally sequentially responsive ESMPs via a side–chain covalent functionalization strategy, effectively improving component compatibility and cyclic stability. The introduction of rigid Azodm units did not induce uncontrolled polymerization or unwanted cross–linking, but is hypothesized to provide a distinct steric hindrance effect, offering a reliable molecular design approach for precisely and linearly tuning the matrix T_g_. Despite a slight decrease in tensile strength, the improved structural homogeneity ensured that the material maintained its excellent shape fixity (R_f_ > 98%) and recovery (R_r_ > 95%), alongside enhanced cyclic stability. Furthermore, rapid, isothermal UV–induced bending below T_g_ demonstrated that the *trans*–to–*cis* twisting of aligned side chains can effectively drive the coordinated motion of the epoxy main chains. However, spontaneous de–alignment over time may still limit the material’s long–term shape stability. Therefore, future work will focus on stabilizing the aligned state and developing multi–wavelength, orthogonally reconfigurable networks to eliminate spectral crosstalk, ultimately advancing soft robotics, smart aerospace morphing structures, and flexible electronics.

## Figures and Tables

**Table 1:** Composition of EPD series samples.

Samples	E–51 (g)	PPGDE (g)	DGA (g)
EPD_99_	7.35	0.1	2.1
EPD_98_	7.27	0.2	2.1
EPD_96_	7.12	0.4	2.1
EPD_94_	6.97	0.6	2.1
EPD_92_	6.82	0.8	2.1

**Table 2:** Composition of EPDm series samples.

Samples	E–51 (g)	PPGDE (g)	DGA (g)	Azodm (g)
EPDm_2_	6.97	0.6	1.995	0.197
EPDm_4_	6.97	0.6	1.89	0.394
EPDm_6_	6.97	0.6	1.785	0.591
EPDm_8_	6.97	0.6	1.68	0.788
EPDm_10_	6.97	0.6	1.575	0.985

## Data Availability

Data are contained within the article.

## Supplementary Materials

The following supporting information can be downloaded at: https://www.mdpi.com/article/10.3390/polym18151902/s1, Table S1: Molecular weight and distribution of EPD_94_ and EPDm_6_; Figure S2: SEM images of EPD series samples: (a) EPD_99_; (b) EPD_98_; (c) EPD_96_; (d) EPD_94_; (e) EPD_92_; Figure S3: SEM of EPDm series samples: (a) EPDm_2_; (b) EPDm_4_; (c) EPDm_6_; (d) EPDm_8_; (e) EPDm_10_; Figure S4. Determination of UV response curvature; Figure S5: Temperature changes of EPDm_10_ surface hotspots during UV response (a)–(f); Equation S6: Evaluation of cyclic shape memory parameters.
