# Supplementary material for: Photo–Thermally Sequentially Responsive Shape Memory Polymers Based on Side–Chain Azobenzene–Functionalized Epoxy
Source: Polymers (Basel). 2026 Aug 3;18(15):1902. doi: 10.3390/polym18151902 (PMC13467885; doi:10.3390/polym18151902)
Supplement: Supplementary file 1 [file polymers-18-01902-s001.zip › polymers-4399044-supplementary.pdf]

Photo-thermally Sequentially Responsive Shape Memory Polymers Based on Side-Chain Azobenzene-Functionalized Epoxy

Jinxiong Wen [1], Xianhao Mao [1], Shaojun Chen [1]*, Yuanyuan Li[1], Zhiwen Tu[1], Huilin Lai[1] and Haitao Zhuo [2]*

Supplementary materials

Table S1. Molecular weight and distribution of EPD_{94} and $EPDm_{6}$.

Samples	Mn	Mw	Mz	Mϱ	D
EPD_{94}	6834	7490	8137	8160	1.096
$EPDm_{6}$	6457	7155	7865	6961	1.108

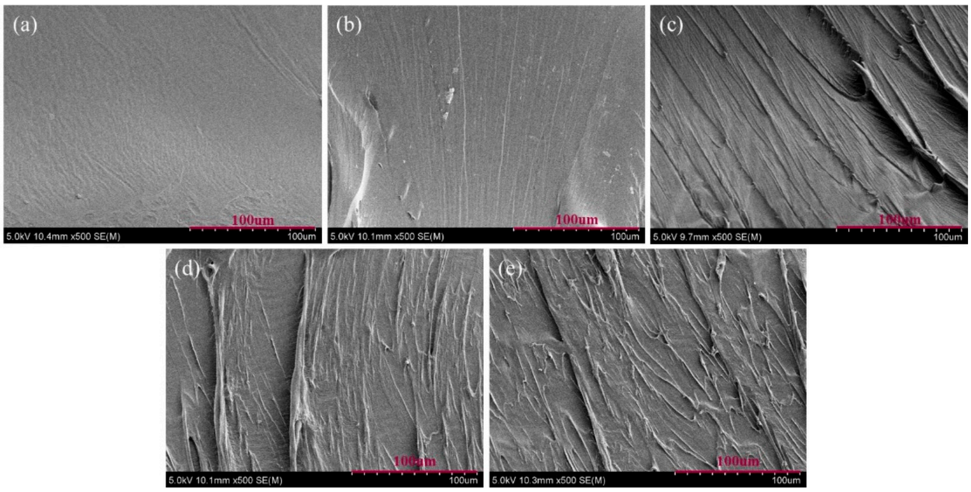

Figure S2. SEM images of EPD series samples: (a) EPD_{99}; (b) EPD_{98}; (c) EPD_{96}; (d) EPD_{94}; (e) EPD_{92}.

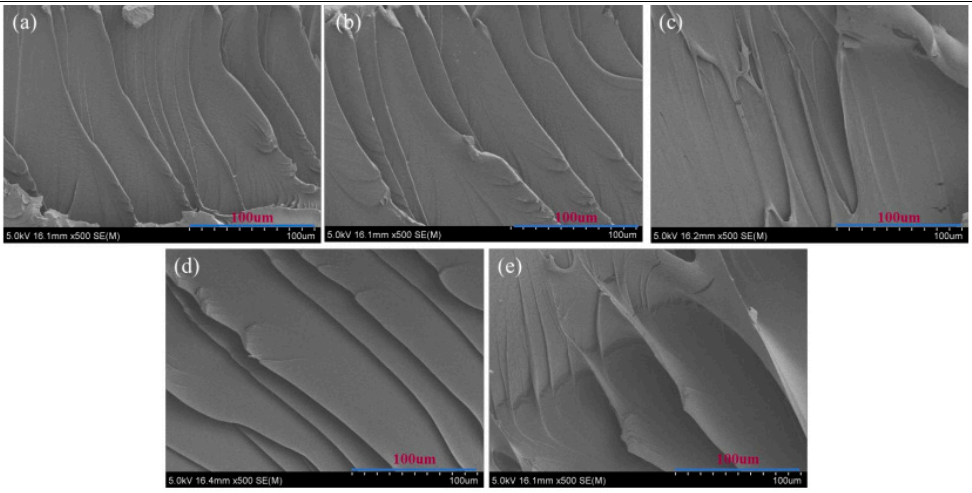

Figure S3. SEM of EPDm series samples: (a) $EPDm_2$; (b) $EPDm_4$; (c) $EPDm_6$; (d) $EPDm_8$; (e) $EPDm_{10}$.

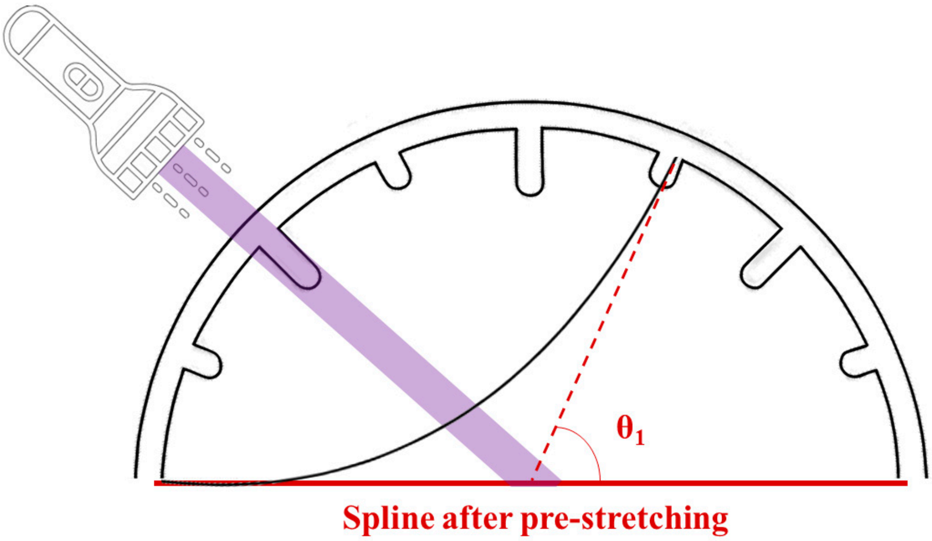

Figure S4. Determination of UV response curvature

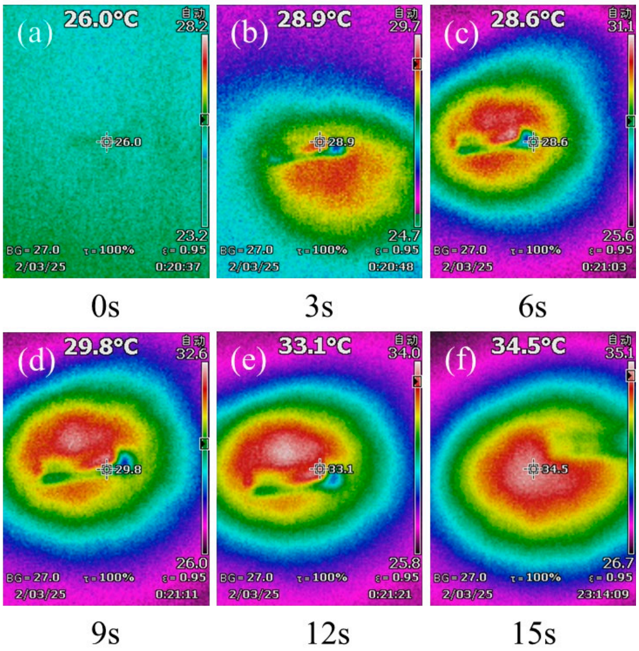

Figure S5. Temperature changes of $EPDm_{10}$ surface hotspots during UV response (a)-(f)

Equation S6. Evaluation of Cyclic Shape Memory Parameters

$$R_f = \frac{\varepsilon_f}{\varepsilon_0} \times 100\%$$

(i)

$$R_r = \frac{\varepsilon_f - \varepsilon_r}{\varepsilon_f} \times 100\%$$

(ii)

$$R_{fn} = \frac{\varepsilon_{fn}}{\varepsilon_{0n}} \times 100\%$$

(iii)

$$R_{rn} = \frac{\varepsilon_{fn} - \varepsilon_{rn}}{\varepsilon_{fn} - \varepsilon_{r(n-1)}} \times 100\%$$

(iv)

Individual shape memory parameters are evaluated via Equation S6 i and ii. For cyclic protocols, R_{fn} and R_{rn} denote the shape fixity and recovery ratios during the n cycle, respectively, where ε_{0n}, ε_{fn}, and ε_{rn} represent the maximum strain under loading, the fixed strain after cooling and unloading, and the residual strain after thermal recovery. Notably, when calculating cyclic parameters (Equation S6 iii and iv), the denominator for R_{rn} requires subtracting the preceding residual strain $\varepsilon_{r(n-1)}$. This correction is essential because the recovery process of the prior cycle has concluded, rendering the accumulated residual strain permanent and irreversible.
